# Gigantic submandibular pleomorphic adenoma: A rare case report

**DOI:** 10.1016/j.ijscr.2019.10.033

**Published:** 2019-10-24

**Authors:** Mohamed Abdelkhalek, Mohamed Elmetwally, Alaa Mazy, Mona Gad, Ahmed Elsaid, Shadi Awny, Basel Refky, Ahmed Abdallah, Amr F. Elalfy, Farida A. Shokeir, Samar Abdallah, Abeer Elfeky, Rowaa Aboelamayem, Mohamed A. Hegazy

**Affiliations:** a Surgical Oncology Unit, Oncology Center Mansoura University (OCMU), Egypt; b Department of Anesthesia and Surgical Intensive Care, Faculty of Medicine, Mansoura University, Egypt; c Department of Pathology, Faculty of Medicine, Mansoura University, Egypt

**Keywords:** Giant, Pleomorphic adenoma, Submandibular gland

## Abstract

**Introduction:**

Pleomorphic adenoma (PA) is the most common benign salivary gland tumor. When neglected on some occasions, its size and weight can enormously augment.

**Case presentation:**

We report a case of a giant submandibular pleomorphic adenoma in a 75 years old female patient which measured 34 × 26 × 20 cm and weighed 8.1 kg.

**Conclusion:**

Neglecting such tumors without treatment, can cause severe facial disfigurement and can even hinder the airway.

## Highlights

• Pleomorphic adenoma can attain huge sizes if neglected.
• It could cause facial disfigurement and compromise the airway.
• Surgical resection is the main stay of treatment.

## 1 Introduction

PAs or benign mixed salivary gland tumors are the most common neoplasms that arise in both major and minor salivary glands. According to Pinkston 1999, approximately 75%–85% of all PAs occur in the parotid gland, with only 8% arising in the submandibular gland. Those occupying the minor salivary glands represent 7–15% of all reported cases [1].

PAs occur among all age groups, with the incidence rate of about 3.5/100,000. As the age progresses, the incidence rate escalates, but among older individuals; 65–74 years, a decline is noticeable. It is found more common among females [2]. While a majority of such tumors are found in parotid glands (80%), the percentage of those involving submandibular glands is reportedly only (12%). Therefore submandibular PA is considered rare [3,4].

We report this unique case to stress that such tumors can attain gigantic sizes and, if neglected and left untreated, could cause extreme facial disfigurement and also could affect the airways and compromise normal breathing. We report a case, which could be one of the largest recorded PAs of the submandibular gland.

## 2 Case presentation

A 75-year-old woman was referred to our hospital complaining of a huge swelling in the left side of her face and neck. She stated that this mass had begun to develop 15 years ago at which it was only the size of a small almond at the left side of her neck. The mass was painless and gradually increasing in size but the patient refused to get medical consultation throughout these years till she started to feel serious difficulty in breathing.

**Fig. 1:**
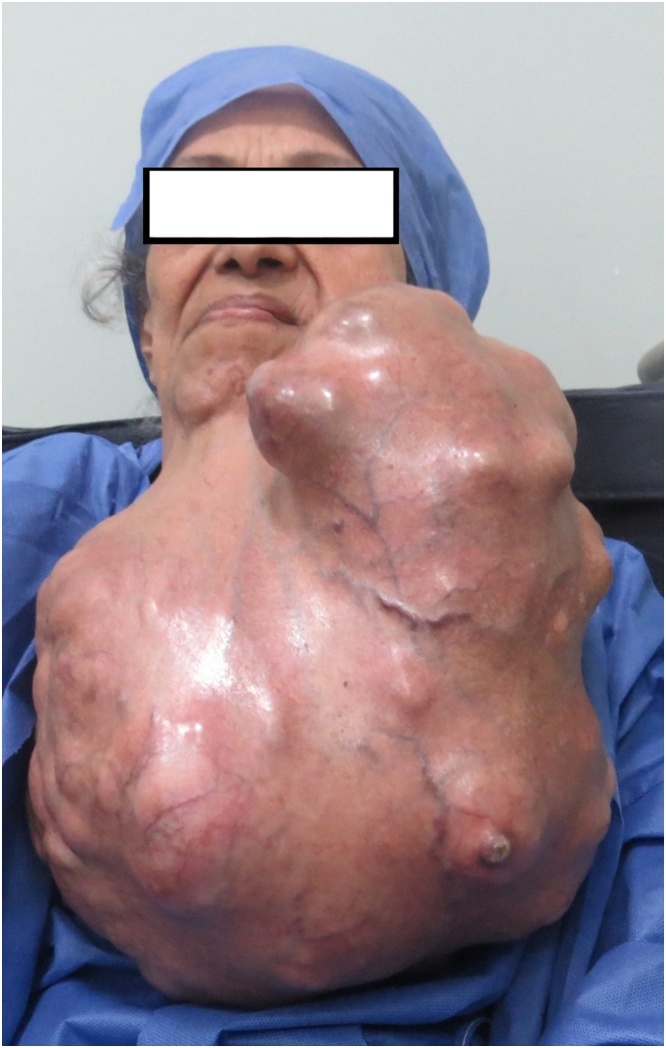
Preoperative antero-posterior view.

**Fig. 2:**
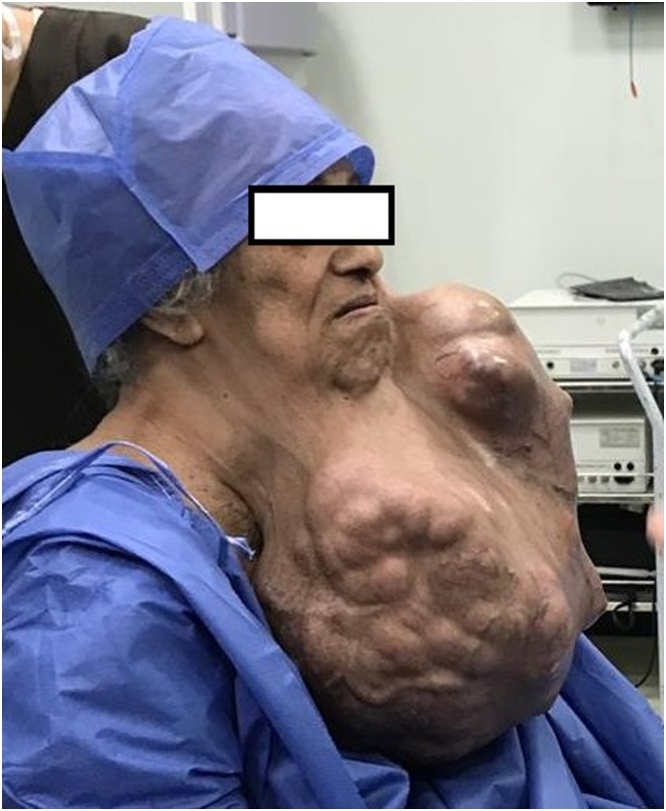
Preoperative lateral view.

Clinical examinations revealed a huge firm mass in the left side of the face and neck crossing the midline reaching the right side of the neck and inferiorly till reaching below the left inframammary sulcus. The mass itself was not tender, with nodular bosselated outer surface and the surrounding skin was congested in some areas (Fig. 1, Fig. 2).

The origin of the mass couldn’t be determined clinically whether it arised from parotid, submandibular or even thyroid gland. A core-needle biopsy suggested a salivary glandular origin.

**Fig. 3:**
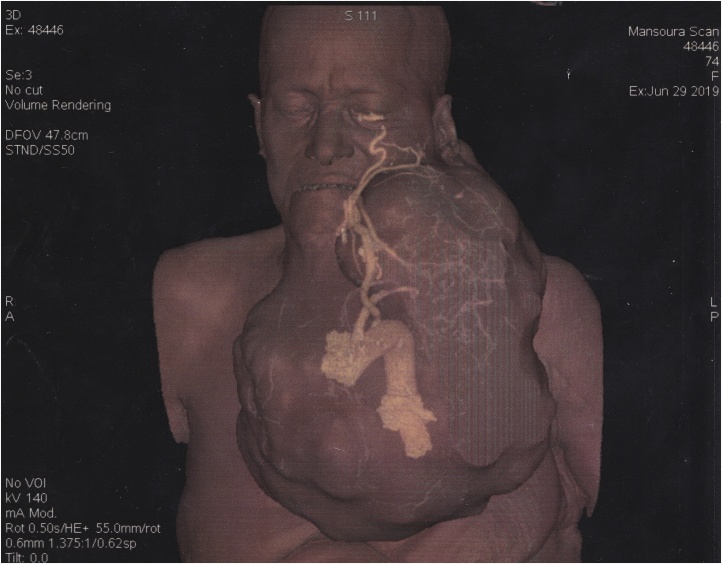
CT angiography (antero-posterior view).

**Fig. 4:**
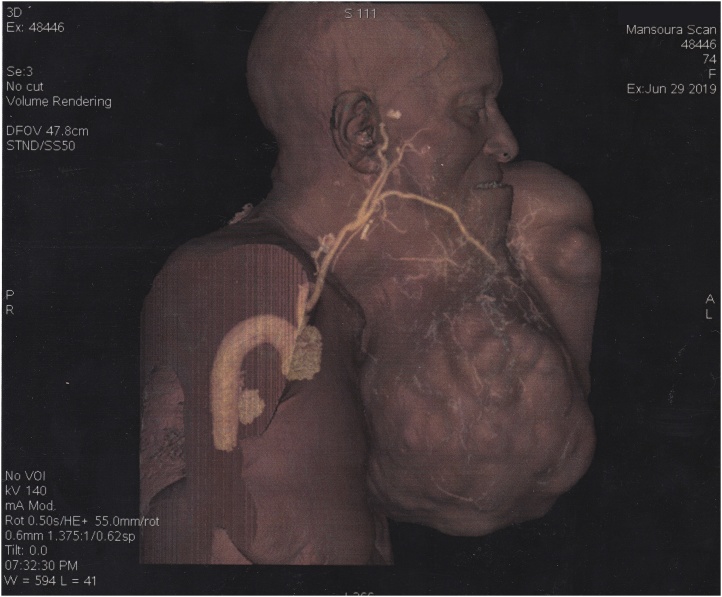
CT angiography (lateral view).

CT scan with contrast of the neck was done and revealed a huge mass (34 × 20 × 26 cm) arising from left side of the neck and extended to parapharyngeal and sublingual spaces with displacement of trachea to the right side. It was found separated from thyroid gland and surrounding structures. CT angiography was also done and showed that the main arterial supply was from the facial branch of the left external carotid artery, common, external and internal carotid arteries on both sided which were patent and of normal shape (Fig. 3, Fig. 4).

**Fig. 5:**
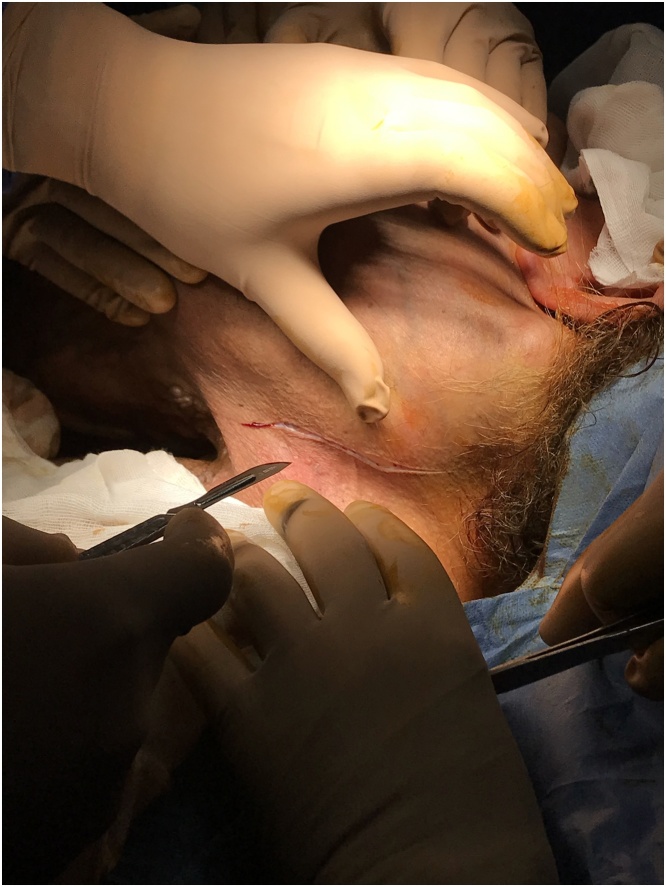
Inferolateral incision.

**Fig. 6:**
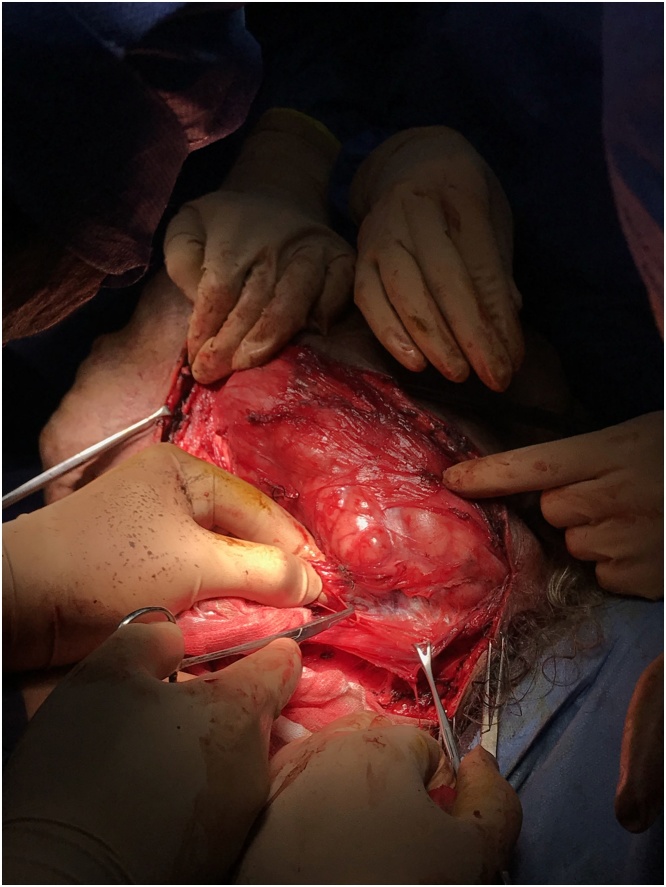
Intraoperative dissection.

**Fig. 7:**
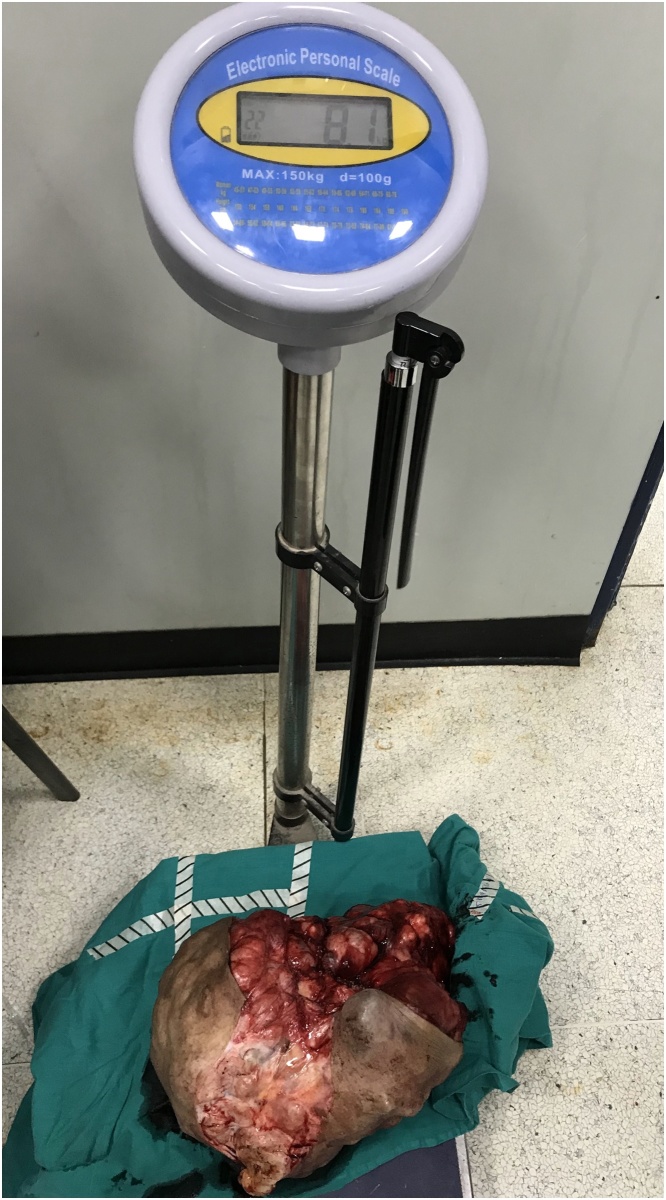
Postoperative specimen.

**Fig. 8:**
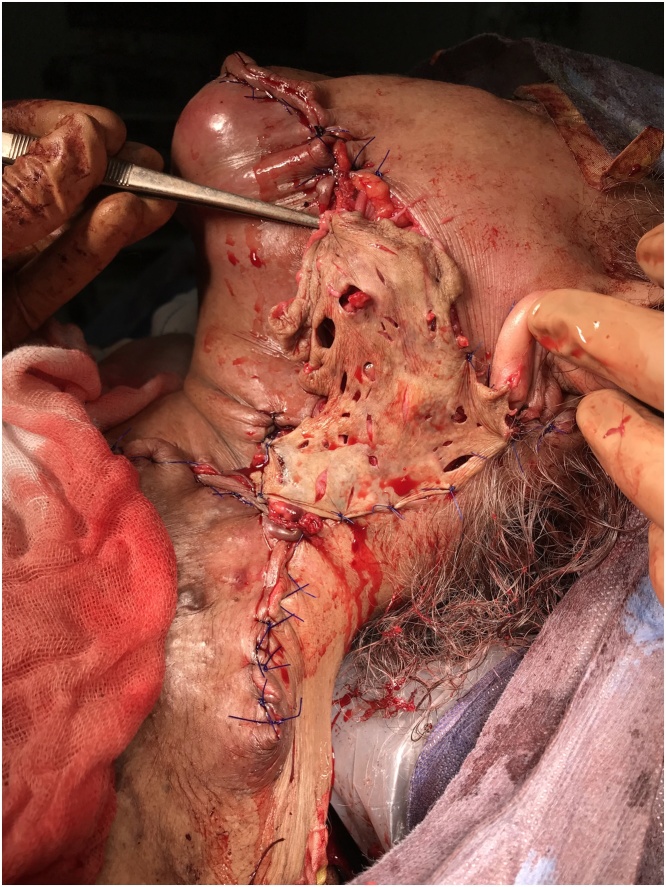
Covering with Thiersch graft.

The patient was prepared for surgery. Anesthesia team used fibreoptic intubation. We started with a left inferolateral incision to separate the tumor from the neck great vessels. After securing them, we continued the dissection both superiorly and inferiorly in the medial direction to the right side (Fig. 5, Fig. 6). There was no evidence of surrounding tissue infiltration. We were able to remove the tumor completely which weighed 8.1 kg (Fig. 7). The tumor had a very wide base therefore the defect couldn’t be closed primarily and we used a thiersch graft (Fig. 8).

**Fig. 9:**
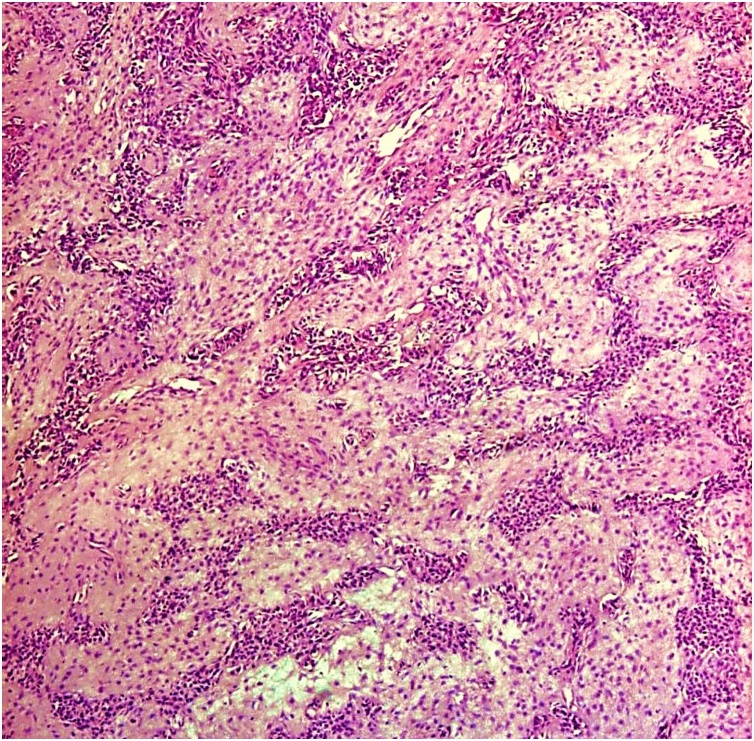
Pleomorphic adenoma shows alternating.

**Fig. 10:**
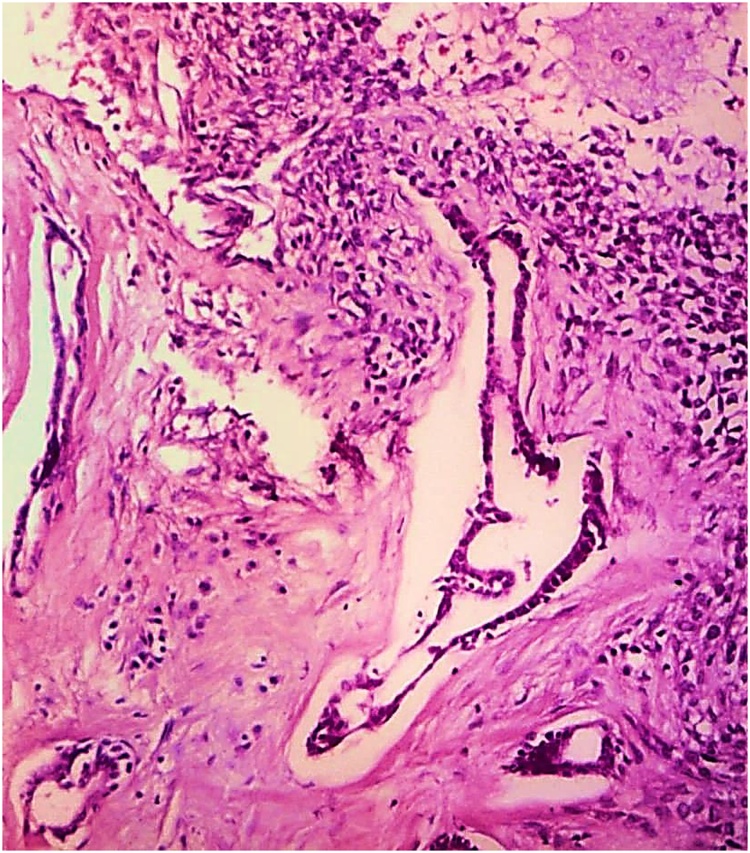
Sheets of epithelial and myoepithelial cells. (H&E. ×40).

**Fig. 11:**
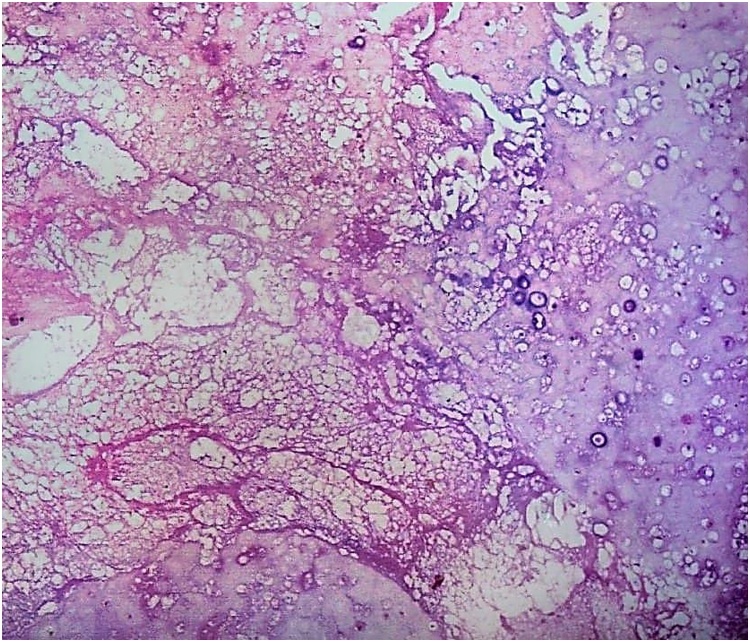
Acinar structures lined by epithelial cells admixed with myo-epithelial cells and myxoid matrix. (H&E. ×100).

**Fig. 12:**
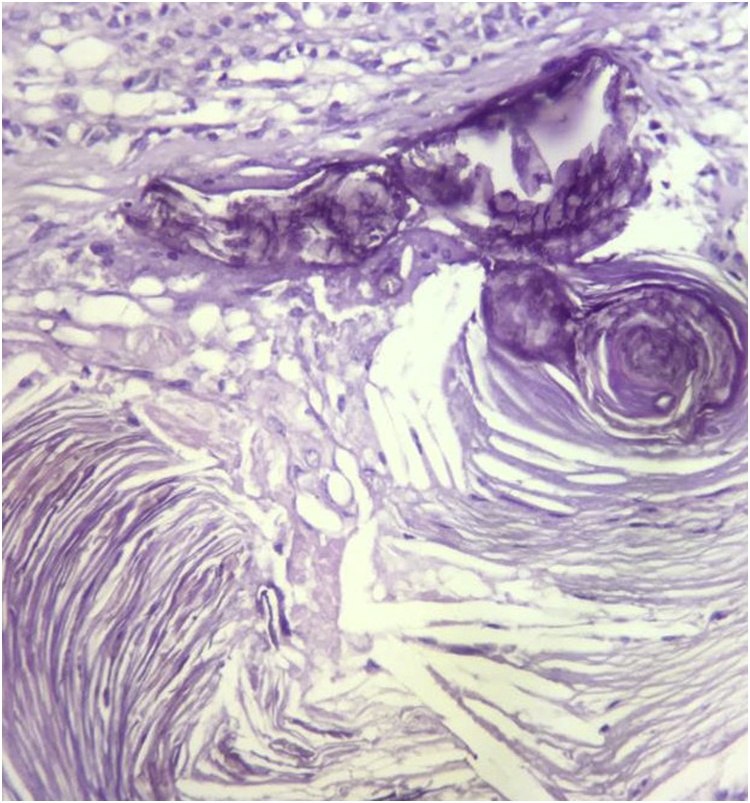
Dystrophic calcification (Hx&E, ×400).

**Fig. 13:**
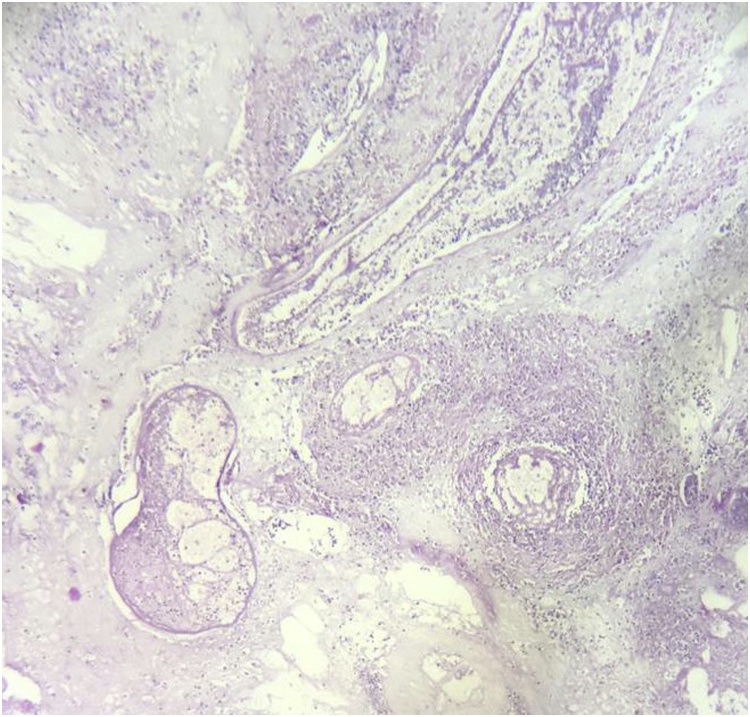
Infarction necrosis exhibiting ghosts of cells (Hx&E, ×40).

**Fig. 14:**
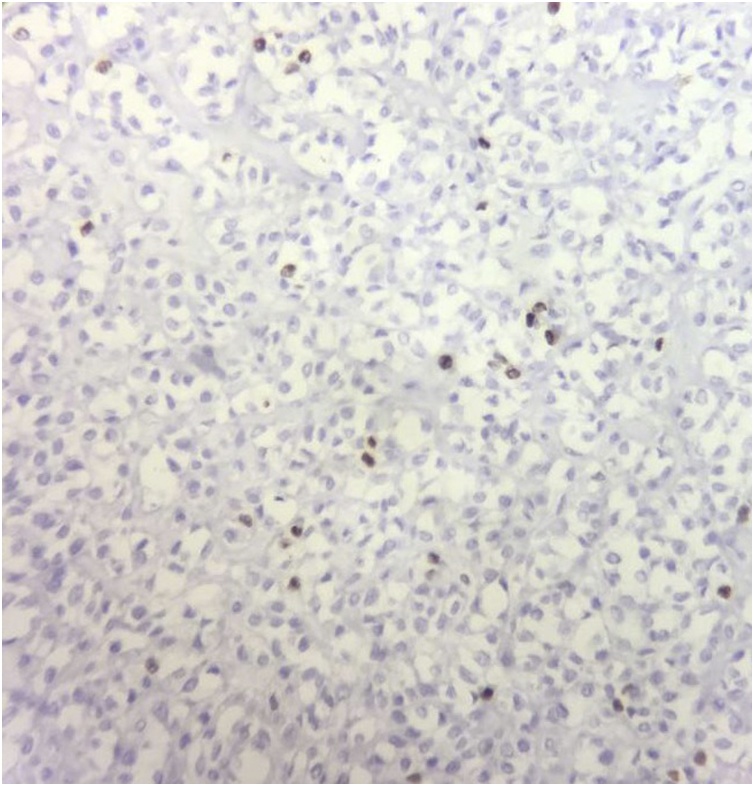
Nuclear positivity of ki67 in 2% of tumor cells (×40).

The histopathology report grossly stated that the mass had lobulated irregular outer surface, and was covered by a skin flap measuring 30 × 10 cm which was grossly unremarkable. Cut section was heterogeneous showing yellowish friable areas admixed with whitish glistening areas, with wide areas of haemorrhage and cystic degeneration. Ten paraffin blocks were prepared. Microscopic analysis yielded a biphasic benign mixed tumoral proliferation formed of epithelial and myoepithelial components that were embedded against a myxochondroid matrix. The epithelial component comprised ductal structures lined by bland looking cuboidal cells. These were associated with clear plumpy, rounded epitheloid and plasmacytoid, as well as spindle shaped myoepithelial cells admixed with cartilaginous foci against myxoid background (Fig. 9, Fig. 10, Fig. 11). Multiple areas of infarction necrosis were seen with ghosts of cells appearing, admixed with areas of dystrophic calcification (Fig. 12, Fig. 13). There was no detected atypia or malignancy in all dissected areas with no evident mitotic figures nor metaplastic elements. The tumor had very low proliferation index as proved by ki67. It exhibited nuclear positivity in only about 2% of tumor cells (Fig. 14).

The patient was discharged three days postoperatively in a good general condition with marvelous improvement of her respiration and facial appearance.

To date, the largest ever recorded PA arising from the submandibular gland was 22 × 25 × 19 cm [5]. Our case measured 34 × 20 × 26 cm and weighed 8.1 kg.

## 3 Discussion

The incidence of giant PA is extremely uncommon, and it mostly arises from the parotid gland. It mainly occurs in females, with a mean weight of approximately 7.8 kg [6]. Gupta et al reported weights of PAs ranging from 1 kg to 27 kg [7].

It is extremely rare to report enormous Pas in the submandibular gland, only a few cases have been reported [7,8]. Being painless and slowly growing, some patients may neglect it to such impressively large sizes especially if surgery was a psychological setback for them.

These tumors are usually painless, smooth, firm and asymptomatic until they attain major sizes mostly in the form of pressure symptoms. Imaging studies such as CT and/or MRI can give a reasonable diagnosis. It is usually well demarcated from the surrounding tissue by a pseudocapsule due to compression of the surrounding parenchyma and fibrosis. If this fibrous capsule is completely excised, these tumors could be cured with surgery. In huge sizes they could develop a variegated appearance with areas of hemorrhage, necrosis and calcification. Giant tumors commonly have a lobulated delineation, which supports the diagnosis.

At the histological level, PAs demonstrate several phenotypes. Epithelial cells are arranged in sheets and islands showing typical ductal structures lined by epithelial cells with surrounding myoepithelial cells with heterogeneous features as spindle, squamous, clear, basaloid, oncocytic and sebaceous. The stroma characteristically is diverse with fibrous, chondroid, myxoid or hyaline aspects [9].

The incidence of malignant transformation in PA ranges from 1.9% to 23.3% [10]. The risk rises in tumors with long history of appearance, recurrences and increased age of the patient. Our patient did not have any malignant features, as the tumor included only benign epithelial and myoepithelial tissues intermixed with myxoid components.

Recurrence of such tumors is possible especially if the surgical margins are involved, that’s why it should be managed through complete excision of the tumor reaching completely free surgical margin.

Presenting such cases highlights the problem of many patients especially in the developing countries who neglect such grave conditions which whatever the reasons should not be pardoned.

## 4 Conclusion

Giant PA of the submandibular gland is a rare finding, and should be diagnosed cautiously. The proper treatment is complete surgical excision reaching free surgical margins.

## Sources of funding

There is no funding from any organization.

## Ethical approval

Case reports are exempted from ethical approval in my institution.

## Consent

Written informed consent was obtained from the patient for publication of this case report and accompanying images. A copy of the written consent is available for review by the Editor-in-Chief of this journal on request.

## Author contribution

Mohamed Abdelkhalek: study design and writing.

Mohamed Elmetwally: data collection.

Alaa Mazy: data analysis.

Mona Gad: data analysis.

Shadi Awny: writing.

Basel Refky: writing.

Ahmed Abdallah: data collection.

Amr F Elalfy: data analysis.

Samar Abdallah: data collection.

Abeer Elfeky: data collection.

Mohamed A Hegazy: supervision.

## Registration of research studies

researchregistry5050.

## Guarantor

Mohamed Abdelkhalek.

## Provenance and peer review

Not commissioned, externally peer-reviewed.

## Declaration of Competing Interest

There is no conflict of interests.
